# From the Editor’s desk

**DOI:** 10.4103/0974-1208.74150

**Published:** 2010

**Authors:** Kamini Rao

**Affiliations:** Editor-in-Chief, Journal of Human Reproductive Sciences E-mail: kambacc@vsnl.com

Welcome to the last issue of the journal for the year 2010, the year that saw the readership base grow as ISAR membership increased from 600 in 2009 to 1015 currently. The journal also has more than 13,000 people accessing the issue online. Scientists, like everyone else, want to publish papers in journals where their work is likely to reach out to as large an audience as possible, thus having the highest impact. The steady increase in our readership base is testimony to the rising popularity of the journal.

The current issue starts with a review article by Dr. Korula George on fertility and age, which highlights the importance of the changing social scenario and increasing demand for fertility services in older women. It is a well-known fact that age of the female partner remains the most important factor predicting the success of infertility treatment. As more and more women are choosing to conceive at a later age, it puts pressure not only on the couple but also on the treating physicians because they are left with limited treatment options. Though tests exist to predict ovarian reserve, there is no way that we can predict IVF success.

The article on seminal zinc concentration and semen parameters highlights the correlation between seminal zinc and sperm motility, viability and count. As there is no single test to predict male fertility potential, assessment of seminal zinc may be used in conjunction with routine semen parameters.

A prospective observational study by M. S. Kamath throws light on predictor factor for pregnancy after COH. Duration of infertility and male factor (TMF) are shown to be negatively correlated with pregnancy outcome. As a sizeable fraction of infertility patients are given IUI as the first-line treatment option, we need to explore further on the factors affecting IUI success rates.

Obesity is an epidemic of the modern world and we need to know the effect of obesity on reproductive outcome. The present study shows no adverse effects of obesity on ART outcome, but results published in the literature have been conflicting.

P. Saxena’s article shows the effect of metformin therapy in PCOS. An improvement in metabolic profile with metformin may prove helpful in managing these patients. As we all know, managing PCOS is like a double-edged sword requiring a very careful approach to prevent OHSS.

Müllerian anomalies are an important cause of repeated pregnancy loss and the article by Priya Selvaraj highlights the importance of Hysteroscopic Septal Resection with Versapoint that is considered to be a safe and an effective approach. New knowledge on post-translational modifications in ER p57 throws light on the molecular process of male infertility. We need to work beyond semen analysis as far as male infertility is concerned, so bio-informatics analysis of ER p57 can be a useful adjunct to semen analysis in the near future.

Last but not the least, all of you must join me in congratulating Dr. Dhiraj Gada for his efforts in bringing IFFS to India. It is a matter of great pride to our country and ISAR to get an international conference of the stature of IFFS to India. We have succeeded in our bid to host the IFFS in 2016 and we must join together to ensure that the conference is a huge success. I conclude with best wishes to all for a happy and prosperous new year.

